# Extraction, purification, characterization, anticoagulant activity, and anticoagulant mechanism of polysaccharides from the heads of *Hypomesus olidus*

**DOI:** 10.1002/fsn3.1360

**Published:** 2019-12-28

**Authors:** Xuan Zhang, Xin‐Tong Ma, Yu Xu, Guo‐Chuan Jiang, Jia‐Lin Zhang, Xue‐Jun Liu, Xiao‐Hui Yan

**Affiliations:** ^1^ College of Food Science and Engineering Jilin Agricultural University Changchun Jilin China; ^2^ Dunhua Market and Quality Supervision Administration Dunhua Jilin China; ^3^ Jilin Light Industry Design and Research Institute Changchun Jilin China; ^4^ Finance and Taxation College Jilin Business and Technology College Changchun Jilin China

**Keywords:** anticoagulant activity, anticoagulant mechanism, extraction and purification, *Hypomesus olidus* head polysaccharides

## Abstract

The aim of this work was to extract, isolate, and purify polysaccharides from the heads of *Hypomesus olidus* and to evaluate their anticoagulant activities and anticoagulant mechanisms. Response surface methodology was used to optimize the extraction conditions for ultrasonic‐assisted extraction of polysaccharides from the heads of *Hypomesus olidus*. The optimal extraction conditions consisted of ultrasonic power of 275 W, ultrasonic time of 50 min, and solid–liquid ratio of 5 ml/g, giving the yield of crude polysaccharides (GYT) of 7.73 ± 0.042%. Three polysaccharide fractions, GYT‐1, GYT‐2, and GYT‐3 were purified from GYT by using DEAE‐cellulose‐52 column and Sephadex G‐100 column for anticoagulant activities. The results showed that two doses (2 and 4 mg/ml) of GYT‐1 and GYT‐3 could significantly prolong (*p* < .01) in partial thromboplastin time (APTT) (2.19 and 2.37 times, 2.22 and 2.44 times, respectively) and thrombin time (TT) (2.39 and 2.46 times, 2.44 and 2.80 times, respectively) compared with normal control. In particular, GYT‐3 had stronger anticoagulant activity than GYT‐1, and it was composed of arabinose, fructose, glucose, and lactose with molar ratios of 0.595:1: 2.026:0.273. However, GYT‐2 had no anticoagulant activity (*p* > .05). In addition, anticoagulation mechanism of polysaccharides from the heads of *Hypomesus olidus* (GYT‐3) was evaluated. The results showed that the anticoagulant activity of GYT‐3 was based on their binding with antithrombin AT‐III. And the inhibitory effects of GYT‐3 on factor IIa and Xa were related to the concentration of AT‐III in plasma. This study may provide a new and promising anticoagulant drug.

## Graphical abstract

The aim of this work was to extract, isolate, and purify polysaccharides from the heads of *Hypomesus olidus* and to evaluate their anticoagulant activities and anticoagulant mechanisms. The results showed that GYT‐1 and GYT‐3 had significant anticoagulant activities and the anticoagulant activity of *Hypomesus olidus* head polysaccharides was based on their binding with antithrombin AT‐III. This study may provide a new and promising anticoagulant drug.

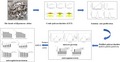

## 1 INTRODUCTION

Cardiovascular diseases such as myocardial infarction, thrombosis, and stroke are main causes of human death, which seriously threaten human health (Kang et al., 2015; Pawlaczyk‐Graja, 2018). Heparin, used in the treatment of cardiovascular diseases and prevention of thrombosis, is the most commonly used anticoagulant drug (Croles et al., 2019; Hao, Xu, Yu, & Zhang, 2019; Kawano et al., 2019). But it could produce side effects such as bleeding and thrombocytopenia (Fu, Suflita, & Linhardt, 2016; Risch, Fischer, Herklotz, & Huber, 2004; Roberts and Susan, 2018). Therefore, it is necessary to discover new and safe anticoagulant active ingredients without the risk of bleeding from natural products.

In recent years, plant‐ and animal‐derived polysaccharides have attracted wide attention because of their strong anticoagulant activities (Song et al., 2019; Wang, Zhang, et al., 2018; Wang, Li, et al., 2018). *Hypomesus olidus*, a freshwater fish, is mainly distributed in northeastern China, Korea, Japan, and Northern Canada (Cho et al., 2006). Because of its fresh aroma of fresh cucumber, it is also known as “cucumber fish”. The fish meat of *Hypomesus olidus* is often used as a delicacy, while fish heads are often discarded. However, studies have shown that by‐products of aquatic products contain a variety of active substances, such as protein, polysaccharide, and fatty acid (Jayathilakan, Sultana, Radhakrishna, & Bawa, 2012). They have significant anticoagulant activities (Song et al., 2019), antioxidant activities (Choksawangkarn, Phiphattananukoon, Jaresitthikunchai, & Roytrakul, 2018), and anti‐inflammatory activities. Therefore, the effective utilization of by‐products can not only avoid waste of resources, but also avoid environmental pollution.

In previous studies in our laboratory, other researchers isolated a natural protein from *Hypomesus olidus*, which proved to have very strong anticoagulant activities (Gou, Wang, & Liu, 2016). However, studies on the anticoagulant activities of polysaccharides from the heads of *Hypomesus olidus* have not been reported.

In the current work, we extracted and purified polysaccharides from the heads of *Hypomesus olidus*, obtained three fractions (GYT‐1, GYT‐2, and GYT‐3), and characterized their structures by ultraviolet and infrared spectroscopy. In addition, the anticoagulant activities of three fractions were evaluated by activated partial prothrombin kinase time (APTT), prothrombin time (PT), and thrombin time (TT). Finally, the anticoagulant mechanism of the anticoagulant activities of polysaccharides from the heads of *Hypomesus olidus* was studied. This work may be helpful in improving the utilization value of *Hypomesus olidus* and providing safe anticoagulants.

## 2 MATERIALS AND METHODS

### 2.1 Materials

*Hypomesus olidus* was purchased from the aquatic product market in Changchun, China. After washing, the fish heads were taken, packaged, and stored frozen at −20°C. The fish bodies could be cooked and eaten or used for further food processing and research. DEAE‐52 cellulose, Sephadex G‐100, and standard monosaccharides, including rhamnose, arabinose, fructose, glucose, galactose, and lactose, were purchased from Sigma Chemical Co. Papain and trypsin were obtained from Shanghai Yuanye Biotechnology Co., Ltd. APTT, PT, and TT assay kits (fixed method) were purchased from Shanghai Sun Biotechnology Co., Ltd. Human antithrombin III (AT‐III), human coagulation factor II (F II), and human coagulation factor X (F X) were purchased from Changchun Baijin Biotechnology Co., Ltd. Rabbit anti‐human AT‐III antibody was acquired by Beijing Boaosen Biotechnology Co., Ltd. Other reagents were analytical grade.

### 2.2 Extraction of polysaccharides from the heads of Hypomesus olidus

The fish heads were degreased with petroleum ether and dried by air. The fish heads (100 g) were homogenized with twice the volume of physiological saline, then centrifuged (3,000 r/min, 20 min), and obtained supernatant. Furthermore, using 0.1 mol/L NaOH solution as alkaline solution, the precipitation after centrifugation was alkalinized by ultrasonic‐assisted method using a KQ dual‐frequency digital ultrasonic cleaner (Kunshan Ultrasonic Instrument Co., Ltd). The supernatant was obtained by centrifugation (3,000 r/min, 20 min). The two supernatants were combined, concentrated, alcohol precipitated, centrifuged, and lyophilized to obtain crude polysaccharides from the heads of *Hypomesus olidus* (GYT).

### 2.3 Optimization of extraction conditions of polysaccharides from the heads of Hypomesus olidus

Based on single factor tests (ultrasonic power, ultrasonic time, and solid–liquid ratio), the extraction conditions of polysaccharides from the heads of *Hypomesus olidus* were optimized by response surface methodology. Three factors and three levels of response surface design were used to determine the optimal conditions for ultrasonic‐assisted extraction. With the polysaccharide yield as response value, the whole design consisted of 17 experimental points, including 12 factor points and 5 axis points. The level of coding test factors is shown in Table 1. The best conditions could be predicted according to the following quadratic models (Wang et al., 2019):

(1) $$Y=\sum A_{0}+\sum_{i=1}^{3}A_{i}X_{i}+\sum_{i=1}^{3}A_{\mathrm{ii}}X_{i}^{2}+\sum_{i=1}^{2}\sum_{j=i+1}^{3}A_{\mathrm{ij}}X_{i}X_{j}$$

where *Y* is the dependent variable, and *X_i_* and *X_j_* are the independent variables; *A*_0_, *A_i_*, *A_ii_*, and *A_ij_* are the regression coefficients of the independent variables that were estimated by the model for the intercept, linear, quadratic, and interaction terms, respectively.

**Table 1:** Box–Behnken experimental design and the results

Run number	A/Ultrasonic power/W	B/Ultrasonic time/min	Solid–liquid ratio/(mL/g)	Polysaccharide yield/%
1	−1	−1	0	7.40
2	1	−1	0	6.95
3	−1	1	0	7.04
4	1	1	0	6.80
5	−1	0	−1	6.99
6	1	0	−1	7.14
7	−1	0	1	7.29
8	1	0	1	6.74
9	0	−1	−1	7.28
10	0	1	−1	6.74
11	0	−1	1	6.93
12	0	1	1	6.63
13	0	0	0	7.71
14	0	0	0	7.71
15	0	0	0	7.74
16	0	0	0	7.81
17	0	0	0	7.67

### 2.4 Isolation and purification of GYT

The proteins in GYT were removed by trypsin combined with Sevage reagents. The GYT solution (3 mg/ml) after protein removal was loaded onto DEAE‐cellulose‐52 anion exchange column. Gradient elution was carried out with distilled water, 0.1, 0.2, 0.3, 0.4, 0.5, and 0.6 mol/L NaCl solution at a flow rate of 0.5 ml/min. The obtained fractions were further purified by Sephadex G‐100 column (1.6 cm D × 60 cm H) at a flow rate of 0.5 ml/min. The polysaccharide content was determined by phenol‐sulfuric acid method at 490 nm (Dubois, Gilles, Hamilton, Rebers, & Smith, 1956). The same fractions were collected, concentrated, dialyzed, and lyophilized to obtain purified polysaccharide samples.

### 2.5 Physicochemical properties of purified samples

Total sugar content was determined by phenol‐sulfuric acid method (Dubois et al., 1956). The protein content was assessed by Bradford (1976). The sulfate content was evaluated by barium chloride‐gelatine method with K_2_SO_4_ as the standard (Dodgson & Prices, 1962). With glucuronic acid as standard, the uronic acid content was determined using the method of Blumenkrantz and Asboe‐Hansen (1973) with slight modification. The solubilities of purified polysaccharide samples in distilled water, acid, alkali, and some organic solvents were evaluated by Gao et al. (2015). The purified polysaccharide samples were dissolved in distilled water (1.5 mg/ml), and the pH of the polysaccharide solutions was determined by a pH meter (Mettler Toledo, Shanghai, China).

### 2.6 Structural characterization of purified polysaccharide samples

#### 2.6.1 Ultraviolet analysis

The purified polysaccharide samples were prepared into aqueous solution of an appropriate concentration and scanned in the wavelength range of 190–400 nm using TU‐1901 ultraviolet‐visible (UV) spectrophotometer (Beijing Pu Analysis General Instrument Co., Ltd).

#### 2.6.2 Infrared spectrum analysis

The purified polysaccharide samples (1 mg) were mixed with dry KBr powder (100 mg), grounded and blended, and then pressed. The structure of the purified polysaccharide samples was analyzed in the range of 4000–400 cm^‐1^ using IR‐Prestuge‐21 Fourier transform infrared spectrometer (Lai Chi Electronic Technology Co., Ltd.).

### 2.7 Anticoagulant activity of purified polysaccharide samples

The anticoagulant activities of purified polysaccharide samples in vitro were determined by PUN‐2048B semi‐automatic coagulation instrument (Beijing Pulang New Technology Co., Ltd.). The three indexes (APTT, TT, and PT) were used to measure the anticoagulant activities of polysaccharides. (Sask, McClung, Berry, Chan, & Brash, 2011).

#### 2.7.1 Determination of APTT

The plasma (0.1 ml) was mixed with APTT reagent (0.1 ml), incubated at 37℃ for 5 min, and 0.1 ml of 0.025 mol/L CaCl_2_ solution was added to record the results of hemagglutination analyzer.

#### 2.7.2 Determination of TT

After preheating the plasma for 3 min at 37℃, 0.2 ml plasma was taken out and mixed with 0.2 ml TT reagent at room temperature. The results of hemagglutination test were recorded.

#### 2.7.3 Determination of PT

After preheating the plasma for 3 min at 37℃, 0.1 ml of the plasma was taken out and 0.2 ml of PT reagent was added to record the results of hemagglutination analyzer.

### 2.8 Monosaccharide composition analysis

The monosaccharide composition of purified polysaccharide samples was determined by high‐performance liquid chromatography. The polysaccharide samples (10 mg) were accurately weighed and dissolved in steamed water (5 ml) and mixed with 0.25 ml concentrated HCl. Samples were hydrolyzed in autoclave at 120 ℃ for 4 hr, neutralized to neutral in NaOH solution, and diluted to 10 ml with steamed water after 48 hr of dialysis. Then, LC‐20AT high‐performance liquid chromatography (Shimadzu, Japan) and InertSustain NH_2_ column (4.6 × 250 mm, 5 μm) were used for analysis.

### 2.9 Study on anticoagulant mechanism

#### 2.9.1 Preparation of plasma

The plasma samples were divided into the following groups: (1) normal control: normal saline mixed with plasma 1/4 (v/v); (2) positive control: heparin sodium (0.05 mg/ml) mixed with plasma 1/4 (v/v); (3) lack of AT‐III plasma group: The human antithrombin Ⅲ (AT‐Ⅲ) activity kit was used to detect the content of AT‐Ⅲ in the plasma to determine whether the lack of AT‐Ⅲ plasma was successfully prepared. Rabbit anti‐human AT‐III antibody was added to the normal plasma. After incubation at 37℃ for 10 min, the mixture was centrifuged (3,000 r/min, 20 min). The supernatant was taken. The content of AT‐Ⅲ in plasma was detected with human antithrombin Ⅲ (AT‐Ⅲ) activity kit, until AT‐Ⅲ was not detected, and the lack of AT‐III plasma was obtained. (4) Plasmas with different AT‐III concentrations: Rabbit anti‐human AT‐III antibodies of 0, 2, 4, 8, 16, and 32 μL were added to 300 μL normal plasma. The mixture was centrifuged (3,000 r/min, 20 min) and the supernatant was taken to obtain plasma with different AT‐III concentration (0:300, 1:150, 1:75, 2:75, 4:75, and 8:75 (antibody/plasma, v/v), respectively).

#### 2.9.2 The relationship between anticoagulant activity of purified polysaccharide samples and AT‐III concentration

1. Anticoagulant activity of purified polysaccharide samples in plasma with different AT‐III concentrations

The polysaccharide samples (4.0 mg/ml) were mixed with 1/4 (v/v) of plasma with different AT‐III concentrations. After incubation at 37℃ for 10 min, the mixture was centrifuged (3,000 r/min, 20 min). The APTT and TT indexes of supernatant were determined according to the above methods.

1. Effect of AT‐III on anticoagulant activity of purified polysaccharide samples with different concentrations

Polysaccharide solutions with concentrations of 0.5, 1.0, 2.0, and 4.0 mg/ml were mixed with 1/4 (v/v) the lack of AT‐III plasma. After incubation at 37℃ for 10 min, the mixture was centrifuged (3,000 r/min, 20 min). The APTT and TT indexes of supernatant were determined according to the methods showed in section 2.7.1 and 2.7.2.

#### 2.9.3 Relationship between the effects of purified polysaccharide samples on the activity of coagulation factor IIa and Xa and the concentration of AT‐III

The purified polysaccharide samples with concentrations of 0, 1, 5, 10 mg/ml, and 15 mg/ml were mixed with normal plasma or lack of AT‐III plasma. After incubation at 37℃ for 10 min, the mixture was centrifuged (3,000 r/min, 20 min). The supernatant was taken, and the activities of human coagulation factor IIa and Xa in plasma was determined by human coagulation factor II and X activity assay kits.

### 2.10 Statistical analysis

SPSS statistical software was used for variance analysis, data processing was carried out using Graph Pad Prism 5 software and Origin Pro 7.5 software, and the results were expressed as mean ± standard deviation (*SD*). The acceptable level for statistical significance was *p* < .01.

## 3 RESULTS AND DISCUSSION

### 3.1 Experiments of single factor

#### 3.1.1 Effect of ultrasonic power on polysaccharide yield

**Figure 1:**
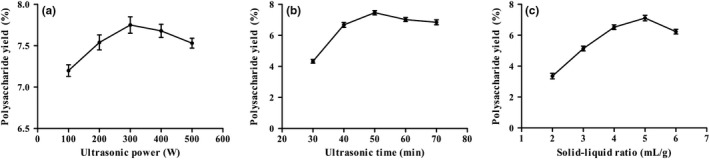
The effect of different extraction parameters on the crude polysaccharide yield from *Hypomesus olidus*. (a) Ultrasonic power; (b) ultrasonic time; and (c) solid–liquid ratio

The effects of different ultrasonic power (100, 200, 300, 400, and 500 W) on polysaccharide yield are shown in Figure 1a. The ultrasonic time and solid–liquid ratio were 50 min and 30 ml/g, respectively. According to Figure 1a, when the ultrasonic power was 300 W, the polysaccharide yield reached the maximum. After this power (300 W), the polysaccharide yield began to decrease. This may be due to the excessive ultrasonic power, resulting in the destruction of polysaccharide structure, thereby reducing the polysaccharide yield.

#### 3.1.2 Effect of ultrasonic time on polysaccharide yield

The effects of different ultrasonic time (30, 40, 50, 60, and 70 min) on polysaccharide yield are shown in Figure 1b. The ultrasonic power and solid–liquid ratio were 300 W and 30 ml/g, respectively. With the increase in ultrasonic time, the yield of polysaccharides increased. When the ultrasonic time was 50 min, the polysaccharide yield was the highest, and then began to decline. This may be due to the degradation of some polysaccharides by too long ultrasonic time (Ying, Han, & Li, 2011; Zhao, Zhang, Li, Dong, & Liu, 2015).

#### 3.1.3 Effect of solid–liquid ratio on polysaccharide yield

The effects of different ultrasonic time (2, 3, 4, 5, and 6 ml/g) on polysaccharide yield are shown in Figure 1c. The other two factors are 300 W and 50 min, respectively. As shown in Figure 1c, the polysaccharide yield increased first and then decreased with the increase in solid–liquid ratio. At 5 ml/g, the polysaccharide yield was the highest. Considering that excessive liquid content could lead to waste and have adverse effects on subsequent concentration process (Wang, Zhang, et al., 2018; Wang, Li, et al., 2018), the solid–liquid ratios of 4, 5, and 6 ml/g were selected as the scope of response surface analysis.

### 3.2 Statistical analysis and model fitting

In response surface model design, each combination experiment was repeated for three times, and the average polysaccharide yield in three experiments was taken as the results. The experimental results in Table 2 were analyzed by using Design Expert 8.0.5 software. The data in Table 2 were fitted by multiple regression, and the regression model equation was obtained as follows:

$$Y=7.73-0.14A-0.17B-0.07C+0.052\text{AB}-0.17\text{AC}+0.06\text{BC}-0.27A^{2}-0.41B^{2}-0.42C^{2}$$

**Table 2:** ANOVA of the regression equation

Sources	Sum of squares	*df*	Mean square	*F*	*p* (Prob > F)
Model	2.52	9	0.28	38.33	<0.0001^a^
A	0.15	1	0.15	20.30	0.0028^a^
B	0.23	1	0.23	31.14	0.0028^a^
C	0.039	1	0.039	5.36	0.0538^b^
AB	0.011	1	0.011	1.51	0.2592^b^
AC	0.12	1	0.12	16.75	0.0046^a^
BC	0.014	1	0.014	1.97	0.2034^b^
A^2^	0.30	1	0.30	41.26	0.0004^a^
B^2^	0.72	1	0.72	98.06	<0.0001^a^
C^2^	0.74	1	0.74	101.66	<0.0001^a^
Residual	0.051	7	7.315 × 10^–3^		
Lack of fit	0.040	3	0.013	4.94	0.0784^b^
Pure error	0.011	4	2.720 × 10^–3^		
Total	2.57	16			

Abbreviations: ANOVA, analysis of variance; *df*, degrees of freedom.

^a^ Significant at *p* < .01,

^b^ Not significant.

From Table 2, it could be seen that the P value was far less than 0.01, that is, the regression model reached a very significant level. The lack of fit (0.0784) was not significant, indicating that the regression equation fits well. The equation regression coefficient value *R* ^2^ (0.9801) showed that the equation could fully reflect the relationship between the response value and the factors. The correction coefficient $R_{\text{Adj}}^{2}$ (0.9545) indicated that the model could explain 95.45% of the variation of response value. Therefore, the model could be used to analyze and predict the extraction conditions of polysaccharides from the heads of *Hypomesus olidus*. In addition, according to Table 2, the linear coefficients (*A* and *B*), the quadratic coefficients (*A* ^2^, *B* ^2^, and *C* ^2^) and interaction coefficient (AC) were at very significant levels, which showed that they had a very significant impact on the response value.

### 3.3 Analysis of response surface

**Figure 2:**
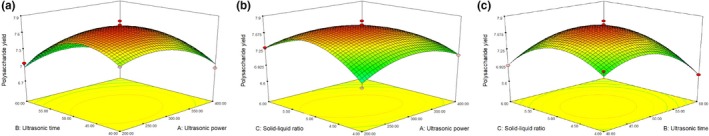
Response surface plots representing the effect of extraction parameters on polysaccharide yield. (a) Ultrasonic power and ultrasonic time; (b) Ultrasonic power and solid–liquid ratio; and (c) ultrasonic time and solid–liquid ratio

3D plots are shown in Figure 2. According to the results of response surface methodology, the optimal extraction conditions of crude polysaccharides from the heads of *Hypomesus olidus* were ultrasonic power 273.82 W, ultrasonic time 47.76 min, and solid–liquid ratio 4.96 ml/g. Under these conditions, the yield of crude polysaccharides was 7.78%. Considering the maneuverability of the experiment, the optimal extraction conditions of polysaccharides were adjusted to 275 W ultrasonic power, 50 min ultrasonic time, and 5 ml/g solid–liquid ratio. Three parallel tests were carried out under the adjusted optimization conditions. The average yield of crude polysaccharides was obtained (7.73 ± 0.042%). The error was 0.64% compared with the theoretical prediction value (7.78%). The results showed that the model was feasible for optimizing the ultrasonic‐assisted extraction conditions of crude polysaccharides from the heads of *Hypomesus olidu* (GYT).

### 3.4 Isolation and purification of GYT

**Figure 3:**
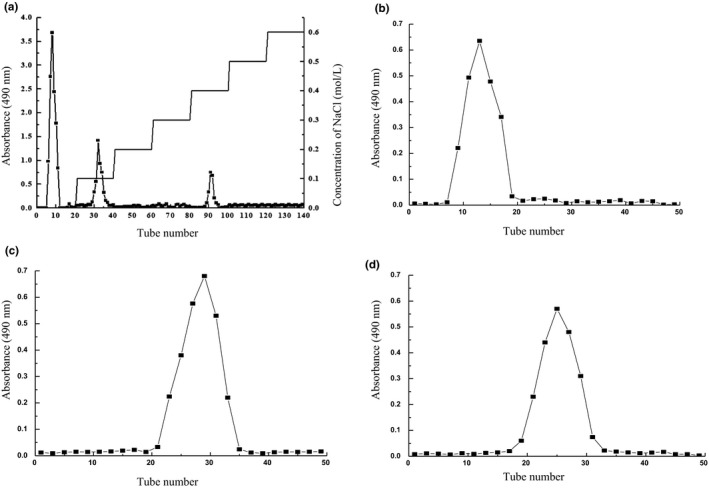
Isolation and purification of crude polysaccharide (GYT) from *Hypomesus olidus*. (a) Elution curve of GYT on DEAE‐cellulose‐52 column; (b–d) Elution curves of Y‐1, Y‐2, and Y‐3 on Sephadex G‐100 column

As shown in Figure 3, GYT was separated by DEAE‐cellulose‐52 column chromatography. Three fractions were obtained by elution with distilled water, 0.1 mol/L NaOH, and 0.4 mol/L NaOH. The three fractions were collected, concentrated, dialyzed, and lyophilized, named Y‐1, Y‐2, and Y‐3 with yields of 536.8 mg/g, 392.7 mg/g, and 276.2 mg/g, respectively. Y‐1, Y‐2, and Y‐3 fractions were further purified by Sephadex column G‐100. All obtained a single component, named GYT‐1, GYT‐2, and GYT‐3.

### 3.5 Physicochemical properties of fractions

In the experiment, it was found that the crude polysaccharides (GYT) were yellow brown powder. The purified polysaccharides (GYT‐1, GYT‐2, and GYT‐3) were all white flocculent powders. GYT, GYT‐1, GYT‐2, and GYT‐3 were all soluble in water. During the experiment, we found that the solubility of GYT decreased with the increase in temperature, while the solubility of GYT‐1, GYT‐2, and GYT‐3 increased with the increase in temperature. This phenomenon may be due to the inclusion of protein and other impurities in GYT. The excessive temperature made the protein denatured to precipitate, resulting in the decrease of its solubility. In addition, the crude polysaccharides and the purified polysaccharides from the heads of *Hypomesus olidus* were soluble in dilute acid and alkali, and were insoluble in organic solvents such as methanol, ethanol, ether, acetone, trichloromethane, and n‐butanol.

The physicochemical properties of GYT, GYT‐1, GYT‐2, and GYT‐3 are shown in Table 3. The total sugar content and protein content of GYT increased significantly (*p* < .01) and decreased significantly (*p* < .01) after purification, indicating that the separation and purification of GYT was effective. In addition, compared with GYT, the contents of glucuronic acid and sulfate decreased significantly (*p* < .01). It was worth noting that GYT‐1 and GYT‐3 contained glucuronic acid and sulfate. And GYT‐3 was significantly higher than GYT‐1 (*p* < .01).

**Table 3:** Physicochemical properties of GYT, GYT‐1, GYT‐2, and GYT‐3. All values are presented as the mean ± *SD* (*n* = 3)

	Total sugar (%)	Protein (%)	Uronic acid (%)	Sulfate (%)	pH
GYT	21.32 ± 0.35	7.30 ± 0.12	17.26 ± 0.17	10.37 ± 0.19	8.24 ± 0.19
GYT−1	81.54 ± 0.56**	0.24 ± 0.01**	2.38 ± 0.11**	3.58 ± 0.08**	7.12 ± 0.14**
GYT−2	80.13 ± 0.62** ^$^	0.15 ± 0.00**	6.87 ± 0.16** ^$$^	1.04 ± 0.06** ^$$^	6.87 ± 0.12**
GYT−3	77.67 ± 0.49** ^$$^	1.32 ± 0.06** ^$$^	9.59 ± 0.21** ^$$^	7.65 ± 0.12** ^$$^	6.38 ± 0.13** ^$$^

* *p* < .05, compared with GYT;

** *p* < .01, compared with GYT;

^$^ *p* < .05, compared with GYT‐1;

^$$^ *p* < .01, compared with GYT‐1.

### 3.6 UV spectrum analysis

**Figure 4:**
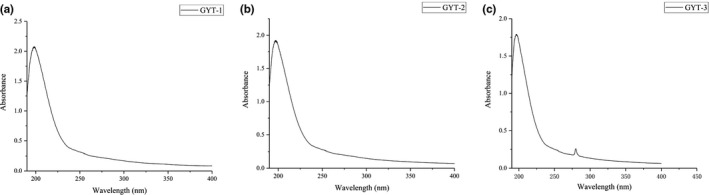
Ultraviolet spectra of GYT‐1 (a), GYT‐2 (b), and GYT‐3 (c)

The UV spectrum of the three components is shown in Figure 4. The UV spectra of GYT‐1 and GYT‐2 were smooth (Figure 4a and b). There were no significant UV absorption peaks at 260 and 280 nm, indicating that GYT‐1 and GYT‐2 contained a trace of nucleic acid and protein. GYT‐3 had no ultraviolet absorption peak at 260 nm, indicating that GYT‐3 contain little nucleic acid. However, its ultraviolet scanning spectrum at 280 nm was prominent, indicating that GYT‐3 may contain proteins binding to polysaccharides, which was in accordance with the results in Table 3.

### 3.7 Infrared spectrum analysis

**Figure 5:**
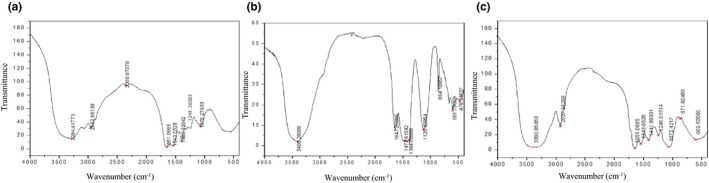
Infrared spectra of GYT‐1 (a), GYT‐2 (b), and GYT‐3 (c)

The composition and structure of each molecule are different, and the energy level transition of molecular vibration is also different in infrared spectroscopy (Abbott, Wolf, Wu, Butterfield, & Kleiman, 1991). According to the characteristic absorption peaks of each functional group in the molecule, its structure can be analyzed and identified. As shown in Figure 5, the characteristic absorption peak of GYT‐1, GYT‐2, and GYT‐3 in infrared spectra of about 3100–3500 cm^−1^, 1870–1650 cm^−1^, and 1400–1200 cm^−1^ was corresponding to the stretching vibration of O‐H, C=O and the angular vibration of C‐H, respectively (Jahanbin, Abbasian, & Ahang, 2017; Xu et al., 2018; Yu et al., 2017). The signal of 1200–1000 cm^−1^ was assigned to the stretching vibration of C‐O‐C, indicating the presence of pyranose ring (Rozi et al., 2019). Infrared spectra proved that there were characteristic absorption peaks of polysaccharides in three fractions.

Moreover, in the infrared spectrum of GYT‐1 and GYT‐3, the absorption vibration of amino group was found near 1543 cm^−1^. The symmetrical stretching vibration of ester bond was observed at 1,411 cm^−1^, indicating the existence of glucuronic acid (Wu, Zheng, Xia, & Kan, 2015). The carboxyl group was also found at 1,400 cm^−1^ in GYT‐1, which proved the existence of glucuronic acid. The signals at 1,244 cm^−1^ and 1,246 cm^−1^ were attributed to the S=O stretching vibration absorption peak (Xu et al., 2019), indicating the presence of sulfate in GYT‐1 and GYT‐3. These results were verified in Table 3.

### 3.8 Anticoagulant activity

#### 3.8.1 Anticoagulant activity of GYT

Three indexes of anticoagulation (APTT, PT, and TT) and their changes were determined by adding different concentrations of GYT in plasma. APTT was used to evaluate endogenous coagulation pathway and PT was used to characterize exogenous coagulation factors (Cai et al., 2016). TT is a thrombin that detects the conversion of fibrinogen to fibrin (Ye, Xu, & Li, 2012). The results are shown in Table 4. Compared with normal control group, GYT with concentrations ≥15 mg/ml could significantly prolong plasma APTT and TT (*p* < .01), indicating that GYT had significant anticoagulant activity. However, there was no significant difference in PT values among all groups (*p* > .05), suggesting that GYT from the heads of *Hypomesus olidus*, achieved anticoagulant effects mainly by inhibiting endogenous coagulation pathway and thrombin‐mediated fibrin formation (Song et al., 2019). Therefore, APTT and TT were used as anticoagulant indicators in the subsequent anticoagulant tests of polysaccharides. The influence on PT was not considered.

**Table 4:** Effects of GYT on plasma coagulation parameters in vitro

Groups (mg/mL)	APTT/s	PT/s	TT/s
Normal control	27.30 ± 1.10	11.27 ± 0.64	12.30 ± 0.75
GYT (5 mg/ml)	28.57 ± 1.10	11.58 ± 0.82	12.64 ± 0.78
GYT (10 mg/ml)	30.43 ± 1.27	11.73 ± 0.73	14.44 ± 0.73
GYT (15 mg/ml)	38.22 ± 1.09**	12.06 ± 0.72	17.53 ± 0.76**
GYT (20 mg/ml)	46.97 ± 1.29**	12.31 ± 0.77	20.47 ± 1.27**
GYT (25 mg/ml)	54.39 ± 1.35**	12.43 ± 0.95	23.77 ± 1.14**

Note

All values are presented as the mean ± *SD* (*n* = 3).

* *p* < .05, compared with normal control group.

** *p* < .01, compared with normal control group.

#### 3.8.2 Anticoagulant activity of GYT‐1, GYT‐2, and GYT‐3

**Figure 6:**
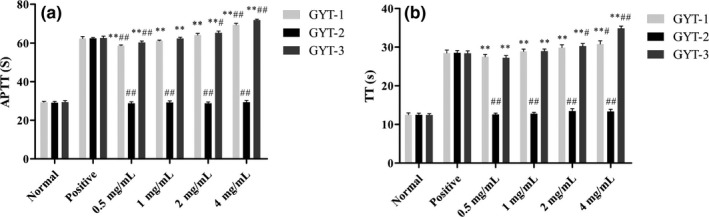
Anticoagulant activity of GYT‐1, GYT‐2, and GYT‐3. The effect of GYT‐1, GYT‐2, and GYT‐3 concentrations on APTT (a) and TT (b) in plasma; All values are presented as the mean ± *SD* (*n* = 3). ^*^ *p* < .05, compared with normal control group. ^**^ *p* < .01, compared with normal control group. ^#^ *p* < .05, compared with positive control group. ^##^ *p* < .01, compared with positive control group

The anticoagulant activities of GYT have been proved. APTT and TT values of three fractions GYT‐1, GYT‐2, and GYT‐3 were determined to verify their activity (Figure 6). Compared with the normal control group, with the increase in GYT‐1 and GYT‐3 concentration, the plasma APTT and TT values in the sample groups increased gradually and showed significant differences (*p* < .01). In fact, compared with the normal control group, at concentrations of 2 and 4 mg/ml, GYT‐1 significantly prolonged (*p* < .01) APTT by 2.19 and 2.37 times, and GYT‐3 by 2.22 and 2.44 times, respectively. Also, at the same dose, GYT‐1 significantly prolonged (*p* < .01) TT by 2.39 and 2.46 times, and GYT‐3 by 2.44 and 2.80 times, respectively, more than that of the control. The anticoagulant activity of GYT‐1 and GYT‐3 with concentrations ≥4 mg/ml was even stronger than that of the positive control group (*p* < .05 or *p* < .01). It was also found that the anticoagulant activities of GYT‐1 and GYT‐3 were in a concentration‐dependent. Furthermore, compared with the normal control group, with the increase in GYT‐2 concentration, APTT and TT in the sample groups had no significant changes (*p* > .05), indicating that GYT‐2 fraction had no anticoagulant activity. This phenomenon indicated that the anticoagulant activity of GYT was due to the presence of GYT‐1 and GYT‐3.

Interestingly, when the concentration was 4 mg/ml, the APTT and TT values of GYT‐3 were significantly higher than that of GYT‐1 (*p* < .01) as shown in Figure 6, indicating that the anticoagulant activity of GYT‐3 was significantly higher than that of GYT‐1. Studies have shown that sulfation of polysaccharides can lead to changes in their biological activities (Wang, Zhang, Yao, Zhao, & Qi, 2013). Sulfate groups play a major role in the antithrombotic activity of heparin (Chen et al., 2015). In this experiment, the results of physicochemical properties have proved that the sulfate content of GYT‐3 was much higher than that of GYT‐1. Therefore, it may be reasonable that the anticoagulant activity of GYT‐3 was higher than that of GYT‐1.

### 3.9 Monosaccharide composition analysis

The monosaccharide composition of GYT‐3 was analyzed by HPLC, and the monosaccharide composition was obtained by comparing with the peak time of standard monosaccharide. As shown in Table 5, GYT‐3 was composed of arabinose, fructose, glucose, and lactose with molar ratios of 0.595:1: 2.026:0.273, respectively.

**Table 5:** Monosaccharide composition of GYT‐3

Standard monosaccharide	Retention time of standard monosaccharides (min)	Retention time of GYT‐3 (min)
Rhamnose	7.557	–
Arabinose	9.285	9.306
Fructose	10.015	10.043
Glucose	10.960	11.002
Galactose	11.628	–
Lactose	18,962	19.021

### 3.10 Anticoagulant mechanism of GYT‐3

#### 3.10.1 The relationship between anticoagulant activity of GYT‐3 and AT‐III concentration

1. Anticoagulant activity of GYT‐3 in plasma with different AT‐III concentrations

**Figure 7:**
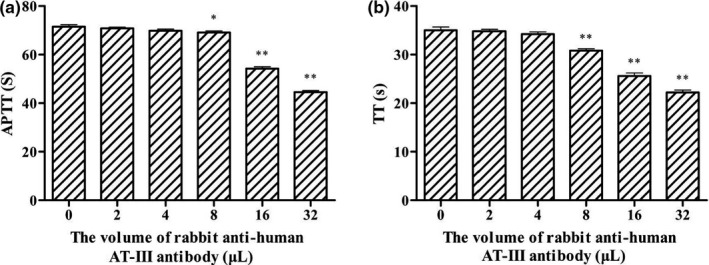
Effect of GYT‐3 on APTT (a) and TT (b) in plasma with different AT‐III concentrations. All values are presented as the mean ± *SD* (*n* = 3). ^*^ *p* < .05, compared with normal plasma. ^**^ *p* < .01, compared with normal plasma

Because of the highest anticoagulant activity of GYT‐3, we used GYT‐3 as raw material to study the anticoagulant mechanism of anticoagulant polysaccharides from the heads of *Hypomesus olidus*. Figure 7 showed that the anticoagulant activity of GYT‐3 decreased with the increase in AT‐III antibody concentration (i.e., with the decrease in AT‐III concentration in plasma). Compared with normal plasma, APTT and TT of GYT‐3 decreased significantly (*p* < .05 or *p* < .01) when 8 μL AT‐III antibody was added to plasma. When the content of AT‐III antibody in plasma was above 16 μL, there was a significant difference in APTT between the experimental group (*p* < .01), indicating that the anticoagulant activity of GYT‐3 was dependent on the concentration of AT‐III in plasma.

1. Effect of AT‐III concentration on anticoagulant activity of GYT‐3

**Figure 8:**
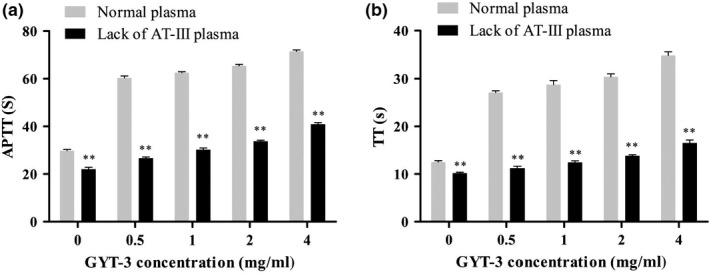
Effects of AT‐III concentration on APTT (a) and TT (b) of GYT‐3. All values are presented as the mean ± *SD* (*n* = 3). ^*^ *p* < .05, compared with normal plasma. ^**^ *p* < .01, compared with normal plasma

The anticoagulant activity of GYT‐3 in lack of AT‐III plasma was significantly lower than that in normal plasma. APTT and TT in lack of AT‐III plasma were significantly lower than those in normal plasma (*p* < .01) (Figure 8a and b). Even if the concentration of GYT‐3 increased from 0 mg/ml to 4 mg/ml, the anticoagulant effects were still not obvious, which further indicated that the anticoagulant activity of GYT‐3 was related to the concentration of AT‐III in plasma. That is, the anticoagulant effect of GYT‐3 was achieved by combining with antithrombin AT‐III.

#### 3.10.2 Relationship between the effects of GYT‐3 on the activity of coagulation factor IIa and Xa and the concentration of AT‐III

**Figure 9:**
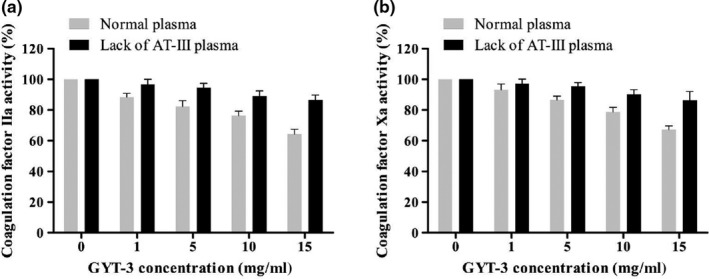
Effect of GYT‐3 on the activity of coagulation factor IIa (a) and Xa (b). All values are presented as the mean ± *SD* (*n* = 3). ^*^ *p* < .05, compared with normal plasma. ^**^ *p* < .01, compared with normal plasma

The influence of GYT‐3 on inactivation of factor IIa and Xa by AT‐III were verified (Figure 9). With the increase in GYT‐3 concentration, the activities of coagulation factor IIa and Xa in normal plasma gradually decreased, indicating that the addition of GYT‐3 in normal plasma had effective inhibitory effects on the activities of coagulation factor IIa and Xa. However, in lack of AT‐III plasma, with the increase in GYT‐3 concentration, the activities of coagulation factor IIa and Xa decreased slightly, but the changes were not obvious. This indicated that the effect of GYT‐3 on coagulation factor IIa and Xa was related to the presence of AT‐III in plasma.

It is reported that heparin is a highly sulfated glycosaminoglycan (Zhi et al., 2018). The anticoagulant effect of heparin is based on its combination with antithrombin AT‐III (Sasisekharan & Venkataraman, 2000), to form a heparin–AT‐III complex, which can inactivate coagulation factors (Ⅱa, Ⅸa, Ⅹa, Ⅺa, and Ⅻa). In this experiment, the infrared spectra of GYT‐3 showed that the functional group composition of GYT‐3 was similar to that of heparin, so the study of anticoagulant mechanism of GYT‐3 was carried out according to the anticoagulant mechanism of heparin. In this experiment, we studied the relationship between the anticoagulant activity of GYT‐3 and AT‐III concentration and the relationship between AT‐III concentration and the activity of coagulation factor IIa and Xa. The results showed that the anticoagulant effect of GYT‐3 was based on their binding with antithrombin AT‐III and was dose‐dependent. At the same time, the inhibition of GYT‐3 on coagulation factor IIa and Xa was also related to AT‐III concentration. The mechanism of action was similar to that of heparin in anticoagulation (Chuang, Swanson, Raja, & Olson, 2001). These results could preliminarily validate the previous hypothesis. These results are also helpful in designing meaningful heparin mimetic molecules to develop safe anticoagulants (Ma et al., 2018).

## 4 CONCLUSIONS

The polysaccharides were extracted from the heads of *Hypomesus olidus* by ultrasonic method; the optimal conditions were 275 W ultrasonic power, 50 min ultrasonic time, and 5 ml/g solid–liquid ratio, giving the yield of crude polysaccharides (GYT) of 7.73 ± 0.042%. The results showed that the purified fractions GYT‐1 and GYT‐3 could significantly inhibited coagulation through intrinsic and common pathways. GYT‐3 with higher anticoagulant activity was composed of arabinose, fructose, glucose, and lactose with molar ratios of 0.595:1: 2.026:0.273. The results of anticoagulation mechanism showed that the anticoagulant activity of *Hypomesus olidus* head polysaccharides was based on their binding with antithrombin AT‐III. In addition, the inhibitory effects of GYT‐3 on factor IIa and Xa were related to the presence of AT‐III in plasma. As a promising anticoagulant polysaccharide, this study provides a theoretical basis for the development of new anticoagulants and functional foods.

## CONFLICT OF INTEREST

All authors declare no conflicts of interest.

## ETHICAL STATEMENT

This study does not involve any human or animal testing.

## INFORMED CONSENT

Written informed consent was obtained from all study participants.
